# Woven Through Time: Five Strategies for Enduring Indigenous-Academic Co-Production

**DOI:** 10.1007/s12237-026-01736-z

**Published:** 2026-06-03

**Authors:** Alyssa A. Outhwaite, Francisca N. Santana, Georgie V. Ferguson, Cherie Matherne, Donald Dardar, Matthew B. Bethel

**Affiliations:** 1 Louisiana Sea Grant, Louisiana State University, Baton Rouge, LA 70803 USA; 2 School of Environmental and Forests Sciences, University of Washington, Seattle, WA 98195 USA; 3 Pointe-au-Chien Indian Tribe, Montegut, LA 70377 USA

**Keywords:** Long-term co-production, Indigenous knowledge, Academic-Indigenous partnerships, coastal resilience

## Abstract

Co-production is increasingly recognized as a transformative approach to addressing environmental and societal challenges; however, key questions remain about how academic and Indigenous partnerships adapt and endure. Existing co-production frameworks offer insights on how to establish new relationships and suggest that long-term partnership may be key to climate adaptation efforts, yet few studies report on the application of co-production to long-term resilience. This perspective explores this gap, reporting on more than a decade of collaborative work between the Pointe-au-Chien Indian Tribe (PACIT) and Louisiana Sea Grant; a partnership rooted in shared goals of community resilience and climate adaptation in coastal Louisiana. Drawing on a basket weaving metaphor, we examine long-term co-production as a process of interlacing diverse knowledge systems, relationships, and strategies into a durable, adaptive structure. While challenges such as institutional power imbalances, environmental disasters and ongoing hazards, and shifting priorities persist, we present these not as obstacles but as opportunities to strengthen the relationship. From this case study, we identify five main strategies that have sustained the PACIT–Sea Grant partnership: (1) Maintaining Flexibility, (2) Evolving Partnerships, (3) Embracing Pluralism, (4) Investing Time, and (5) Building Capacity. We also offer transferable lessons for others engaged in environmental co-production with Indigenous communities. This work contributes to specific strategies and examples on how to build long-term, equitable collaborations in the face of ongoing climate and institutional change.

## Introduction

Co-production offers a transformative way to address complex societal and environmental challenges by rethinking how knowledge is created and applied (Turnhout et al., 2020). Here we define co-production as the process of facilitating connections between researchers, community members, and practitioners to jointly develop knowledge, with the goal of making science more actionable and culturally-relevant while also promoting societal change (Bremer & Meisch, 2017; Chambers et al., 2021). We consider that co-production expands on collaboration models, by including partner perspectives at all stages in the process of knowledge development rather than at a single stage. Co-production is particularly valuable in coastal communities because of the unique environmental changes they face, from acute disasters like hurricanes to chronic changes such as sea level rise and coastal erosion, which require both immediate response and long-term adaptation planning. By drawing on diverse knowledge systems and providing the foundation for intensive collaboration, co-production is a promising framework for coastal community adaptation.

Although co-production can present opportunities for research partnerships with Indigenous communities, missteps and mistakes are common in this work. It has been shaped by longstanding power disparities, histories of knowledge extraction, and failures to obtain informed consent. Historical and ongoing power disparities have been well-documented in environmental research involving Indigenous communities, creating ongoing barriers to equitable collaboration (Bohensky et al., 2013; Granderson, 2017). For example, in some cases, Traditional and Local Knowledge (TLK) about coastal ecosystems has been shared without attribution, undervalued in decision-making, or applied without community consent (Domingue, 2022; Maldonado, 2014). However, through models of co-production, Indigenous knowledge may be held in equal standing with Western scientific approaches while also supporting data sovereignty. These forms of respect and protection of rights build an environment of trust in which Indigenous partners are more willing to share insights for adaptation, which Western scientific approaches alone cannot identify (Berkes, 1993; Latulippe & Klenk, 2020). Co-production with Indigenous groups, therefore, offers the potential to develop and implement more community-driven and culturally-relevant coastal adaptation solutions, such as targeted restoration initiatives via decision support tools identifying vulnerable wetlands that impact Indigenous communities (Bethel et al., 2011). Realizing this potential requires strong and dynamic collaborations, yet sustaining these relationships over long time periods is both challenging and understudied.

Existing co-production best practices typically focus on how to establish partnerships and provide frameworks for equitable collaboration, in relatively short-term settings. Drawing on sustainability research with Indigenous communities, Tengö et al. (2017) highlight the importance of bridging knowledge systems and offer five key strategies for co-producing knowledge. The authors emphasize that creating space to hear from Indigenous communities, confirming understanding of the information produced, and generating and applying products to address ongoing challenges are strategies that best support equitable engagement of Indigenous partners. Bridging knowledge systems is further examined by Reid et al. (2021) in respect to Indigenous collaboration for Canadian fisheries management. This work emphasizes a ‘Two-Eyed Seeing’ framework for knowledge co-existence, where both Indigenous knowledge and Western scientific approaches are equally valued and applied. Reid and colleagues highlight this pathway to co-existence is essential for a sustainable and equitable future. Similarly, based on research in the Arctic, Yua et al. (2022) provide a framework of concentric rings that also centers co-produced knowledge systems, supported through nine actions needed in the research process and nine tools that build equity. Together these collaboration frameworks underscore the importance of Tribal input at all stages, from project development to evaluation, which are critical to the project’s success. Missing from this discourse is how the proposed strategies are applied in long-term adaptation efforts.

While this guidance is critical, especially for researchers and communities looking to start new partnerships, co-production continues to face significant challenges in long-term practice. For example, the difficulties of continued engagement under shifting community priorities, limits on community capacity, and a lack of sustained funding limits the long-term sustainability of co-production (Nadasdy, 1999; Turnhout et al., 2020). A number of studies have illuminated these challenges, showing how conflicting priorities among partners leads to tensions that stymie collaboration (Chambers et al., 2021; Kolesar et al., 2024), and short-term funding cycles can further impede the meaningful engagement that demands substantial time and resources (Morris et al., 2024). Less scholarly attention has focused on what sustains relationships over extended periods or how partnerships navigate significant disruptions like environmental disasters, institutional changes, and/or shifting priorities.

To address this gap in the co-production literature, this perspective explores over a decade of collaboration between Louisiana Sea Grant (Sea Grant) and the Pointe-au-Chien Indian Tribe (PACIT or “Tribe”), a partnership that developed from a shared vision for community resilience addressing ongoing flooding and coastal erosion. This critical case demonstrates how a partnership can maintain effectiveness and trust while navigating major disruptions, from catastrophic hurricanes and institutional transitions, to changing community needs. We examine how the PACIT-Sea Grant partnership has sustained itself through these challenges and identify five strategies that have enabled its longevity: maintaining flexibility, evolving partnerships, embracing pluralism, investing time, and building capacity. Through this case study, we offer practical recommendations for others engaged in environmental co-production work with Indigenous communities in coastal regions.

## Case Study: The PACIT-Sea Grant Partnership

### Case Study Location and Context

Coastal Louisiana has one of the world’s highest rates of land loss from sea level rise, storms, and coastal erosion (Couvillion et al., 2017). These environmental concerns are further exacerbated by socio-economic disparities, whereby some of the most imperiled communities are the least protected and often excluded from decision making (Hemmerling et al., 2020). This is perhaps best evidenced by the unequal displacement of residents in Louisiana after the substantial flooding and storm damage from Hurricanes Katrina and Rita (Groen & Polivka, 2010). In response to these disasters, the Louisiana Coastal Protection and Restoration Authority (CPRA) was developed to coordinate efforts between local, state, and federal groups to create a Master Plan for coastal resilience. Since its visionary inception in 2007, the Master Plan has evolved to become more inclusive and effective by incorporating co-produced restoration ideas from local communities. For example, a collaborative project in Plaquemines Parish created a network of nature-based solutions addressing community concerns (Hemmerling et al., 2022). The resulting restoration plans were adopted in the Master Plan and demonstrate how co-production can generate benefits beyond the scope of a single project, by providing a path toward project implementation.

While CPRA has been essential to protecting Louisiana’s coast, its efforts can fail to address how the application of the Master Plan applies to rural communities, particularly those at the land-water interface. Co-production efforts that support community resilience through vulnerability mapping and restoration planning have helped to narrow this gap (Bethel et al., 2014; Maldonado, 2014). One such community that has benefitted from this co-produced work, is the PACIT, which has roots in one of the oldest, continually inhabited communities in the United States. Located in Terrebonne and Lafourche Parishes, the Tribe traditionally relied on the natural resources from both land and sea, with livelihoods centered around farming, trapping, hunting, and fishing. However, many of these occupations are no longer viable; erosion of fertile lands has reduced or completely removed the capacity for agriculture, while habitat destruction has reduced wildlife game for hunting and trapping (Ellis, 2023; Munster, 2023). Additionally, these coastal areas are subject to high rates of subsidence, which has resulted in marsh fragmentation, saltwater intrusion, and loss of culturally significant spaces (Bethel et al., 2022; Ferguson-Bohnee, 2015). Despite these mounting environmental pressures, the PACIT remains committed to adapting in place, drawing on deep cultural roots and a shared place-based identity.

Over the years, the PACIT has collaborated with multiple organizations to advance their adaptation efforts. For example, the Tribe has worked with university researchers and local non-profit organizations on habitat restoration and with locally-based social scientists who study the intersection between vulnerability and environmental risk (Laska, 1990; Laska et al., 2005; Laska & Peterson, 2011). Many of these partnerships have been instrumental in ongoing resilience efforts, yet have also fluctuated over time due to shifts in funding, capacity, and priorities. Some relationships were intended to be short-term, with partnerships dissolving after project goals were achieved. However, among these partnerships, collaboration with Sea Grant has endured. This relationship also started with an end-date in mind, yet early work revealed a shared dedication to preserving Louisiana’s coast. Sea Grant’s mission of addressing coastal challenges and building community resilience aligns with the PACIT’s adaptation priorities, giving rise to a common vision and commitment to each other that would come to sustain this relationship through time.

### Partnership Origins and Development

**Fig. 1:**
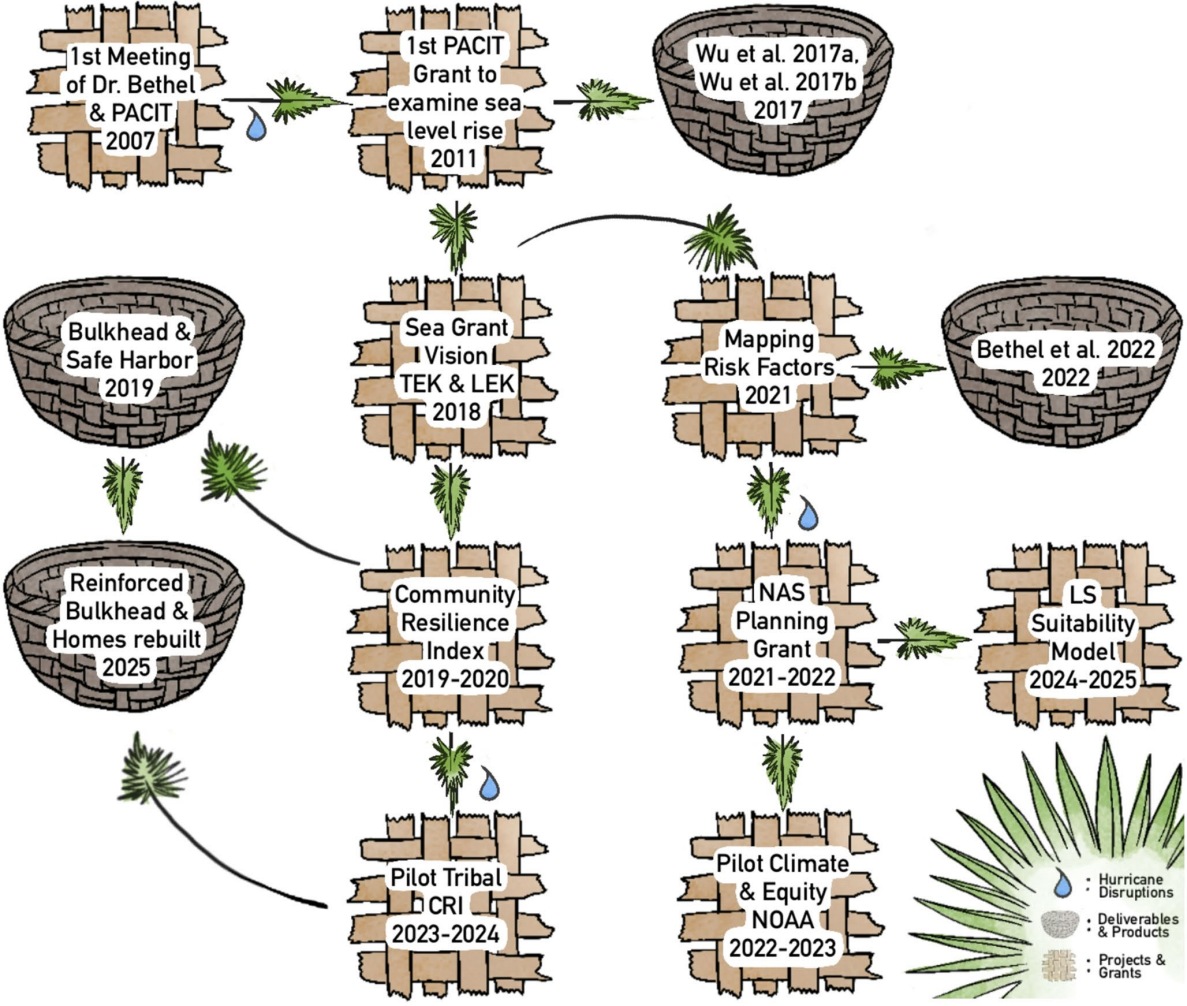
Timeline illustration of Louisiana Sea Grant and Pointe-au-Chien Indian Tribe partnership. Individual projects and grants are represented as woven mats, while project deliverables and products are shown as baskets, and hurricane disruptions are noted with blue raindrops. Image Credit: Kelly Dunn

The partnership between PACIT and Sea Grant began in 2007 (Fig. 1). Drs. Shirley Laska and Kristina Peterson, social scientists with established ties to PACIT, connected the Tribe with then University of New Orleans Ph.D. student Matthew Bethel, whose landscape mapping expertise filled a Tribal need. To *‘lead with the need’* would become a cornerstone for engagement in Dr. Bethel’s future efforts with the Tribe. The first meeting was at a cultural event where Tribe members were teaching traditional palmetto basket weaving. “I struggled to make a basket,” Bethel recalls, “so they showed me how to make a woven crawfish instead, which was much easier. Mrs. Theresa told me to bring it back next time I visited. It was my key into the community.” This exchange with Theresa Dardar, a PACIT member, established a foundation for trust that would be built throughout their future collaboration. Although Dr. Bethel and the PACIT did not collaborate on their first funded grant until 2011—a project examining sea level rise impacts to wetlands and risk perception (Wu et al. 2017a, b; Wu et al. 2017a, b) —trust building began right away. Dr. Bethel’s related work with another local Tribe, Grand Bayou Village, brought him in close contact with the PACIT through their shared participation on the First People’s Conservation Council, an Indigenous association dedicated to addressing concerns related to Louisiana’s natural resources in Tribal spaces. Through this work, the PACIT were able to see how Dr. Bethel’s mapping work could be applied to their own resilience efforts. During this time, Dr. Bethel regularly met with PACIT members, visited field sites of concern, and together sought opportunities to fund resilience research. When Dr. Bethel joined Sea Grant in 2013, the partnership expanded significantly through broader institutional support and resources.

Since then, the PACIT-Sea Grant partnership has supported several research projects, publications, and community resilience initiatives (Fig. 1). Key outcomes from the partnership include establishing a partially funded PACIT Resilience Coordinator position and completing infrastructure improvements identified through a 2018 Community Resilience Index workshop. The partnership has also produced institutional guidance documents and peer-review publications outlining best practices for Indigenous engagement (Bethel et al., 2022; The Sea Grant Network, 2018). Recent efforts have focused on the co-production of a Living Shoreline Suitability Model to inform nature-based climate solutions, supported by the National Academies of Sciences, Engineering, and Medicine (NAS) Gulf Research Program (GRP).

The partnership between PACIT and Sea Grant offers a unique case study through which we can examine the factors that sustain co-production through hazards and disasters, capacity limitations, and shifting community priorities. To identify these factors, we gathered data from both peer-reviewed and grey literature including project documents, grant reports, and meeting records spanning from 2007 to 2025. Additionally, informal interviews were conducted with individuals from both organizations who have been part of the partnership, including the current Director of Research at Sea Grant, the PACIT Resilience Coordinator, and the PACIT Tribal Affairs Liaison.

## Strategies for Sustained Co-Production

**Fig. 2:**
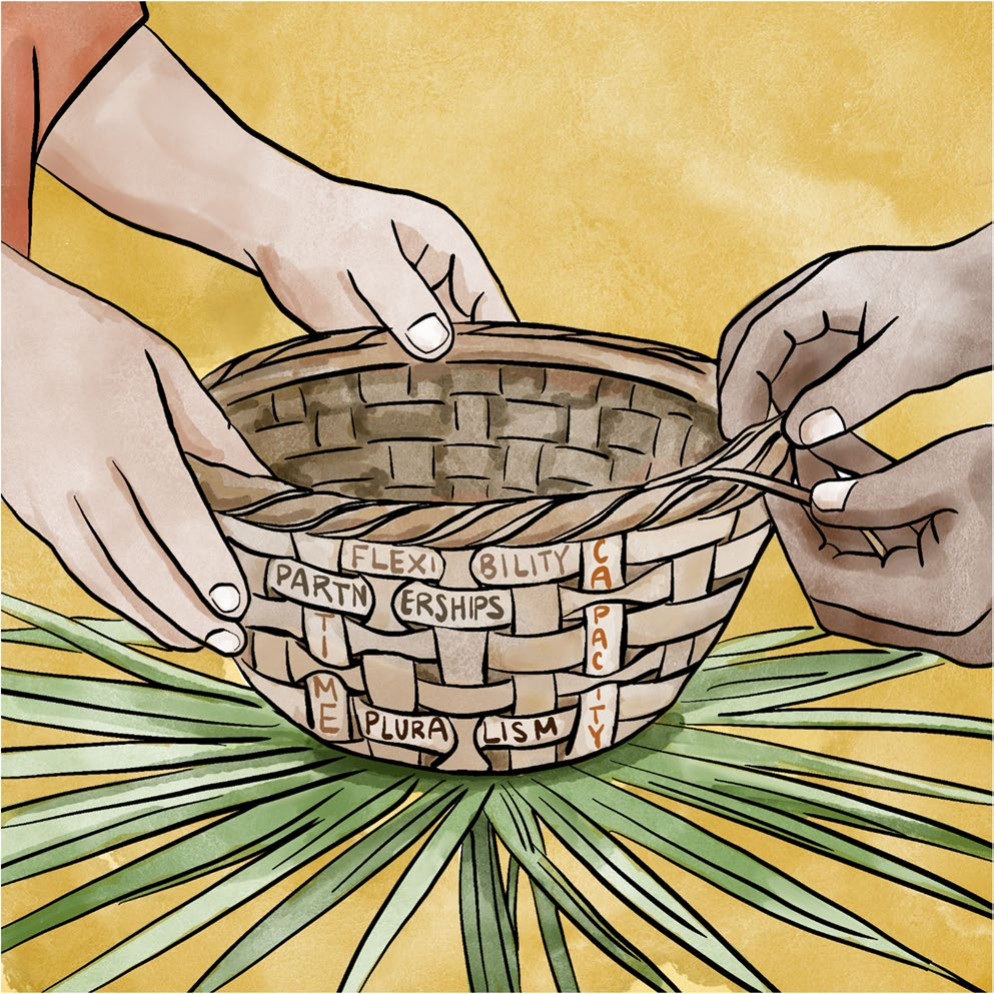
Illustration of co-production strategies, drawing on a basket weaving metaphor, that have made for successful long-term relationships between the PACIT and LSG. Strategies include Flexibility, Partnerships, Pluralism, Time, and Capacity. Image credit: Kelly Dunn

Through an analysis of the PACIT-Sea Grant collaboration, we identify five strategies that have sustained the partnership over more than a decade: Maintaining Flexibility, Evolving Partnerships, Embracing Pluralism, Investing Time, and Building Capacity. The deliberate nature of these strategies reflects the craft found in palmetto basket weaving, a traditional cultural practice that connects the Tribe to their ancestral roots and offers an opportunity for intergenerational knowledge sharing. Every stage of basketry, from the intended use to the specific materials needed, is intentional and purposeful. Palmetto fronds must be cut, dried for several days in the shade, then promptly woven while fibers are still flexible. Younger generations may sit with elders, siblings or even cousins to learn the basics of this process, whoever can spare the time and expertise. These strategies are then used to re-create patterns of the past or form something entirely new, holding the wisdom of the old and newfound patterns side by side in a single weave that draws strength from both. Similarly, engaging in authentic co-production with Indigenous communities requires deliberate strategies that, when intertwined with care, create a strong, resilient vessel capable of holding and sustaining long-term partnership. The PACIT-Sea Grant collaboration demonstrates that while partnerships face real challenges from histories of marginalization and structural inequities, these experiences also invite us to weave differently, with greater intention. Like fibers in a palmetto basket, these strategies interlace into a resilient structure that sustains long-term partnership (Fig. 2). These strategies are not discrete steps, but interdependent practices that become most visible during periods of disruption, constraint, or change.

### Maintaining Flexibility

Sustaining long-term co-production requires maintaining flexible processes that adjust to community rhythms and priorities. We consider that flexibility is a facet of adaptive capacity, where individuals and institutions are able to readily adjust to unexpected situations and take advantage of opportunities as they arise (Armitage et al., 2011; Cinner et al., 2018). Opportunities for these adjustments occur at all stages of engagement, from project planning and field work to navigating major environmental disruptions. In our partnership, we have modified scheduling, meeting agendas, and data collection methodologies to accommodate community needs. As Theresa Dardar reflects, “The relationship has lasted because Sea Grant didn’t come into the community and tell us what to do and where. They listened and let us tell them where we should go.” Many PACIT members balance seasonal work like crabbing and shrimping with cultural events that define community life. Rather than expecting participation in research efforts to conform to rigid institutional calendars, we have learned to plan meetings and workshops around community availability. When there is a need for in-person meetings and field data collection, we have combined activities for efficiency–what we call “dovetailing.”

We have also found that rigid meeting agendas can stifle organic conversations that are more comfortable for Tribal participants when sharing environmental knowledge. During a workshop drawing on Traditional Ecological Knowledge (TEK) of the PACIT, facilitators came prepared with a structured agenda but recognized early on that participants were deeply engaged in discussions that, while tangential to the original plan, were advancing the core objective of identifying priority locations for living shorelines. Facilitators shifted their approach and allowed the conversation to unfold naturally, which preserved the energy and achieved the session’s goals. Flexibility is especially important when partnerships integrate TEK into scientific or planning frameworks, where relational dialogue and story-based communication are often key modes of knowledge transmission (Whyte, 2013).

Flexibility is also essential during major disruptions, like environmental hazards, that can interrupt research projects and put pressure on partnerships. During Hurricanes Ida (2021) and Francine (2024), the PACIT-Sea Grant partnership adjusted project timelines and negotiated project extensions with the NAS GRP, as community priorities shifted to response and recovery from the storms. In these moments, particular attention was paid to community members’ safety and needs before any project concerns. Without these adjustments, the partnership may have fractured under the strain of trying to complete the original project timelines while the community dealt with storm recovery.

The ability to remain flexible and modify processes in response to external pressures and Tribal priorities is critical to partnership longevity. Literature on adaptive capacity emphasizes that systems that can adjust structures and processes are more likely to survive disruptions and remain resilient (Cinner et al., 2018; Granderson, 2017). Academic partners can adopt this model of flexibility by letting Tribal needs determine project goals and engagement structure, whether that means adjusting agendas and deadlines, or the mode in which information is gathered. In collaborative research contexts, this flexibility shows that community partners’ perspectives and needs are valued and encourages sustained participation even when circumstances change or disruptions occur.

### Evolving Partnerships

Long-term partnerships can benefit from strategically welcoming new collaborators as community priorities and project needs change. The PACIT-Sea Grant collaboration demonstrates this evolution across more than a decade. In 2011, Sea Grant facilitated a connection between the Tribe and sea level rise experts from the University of Southern Mississippi to assess coastal vulnerability and evaluate the potential for wetlands to adapt to rising waters. Results from this project directly informed the Tribe’s resilience planning to safeguard cultural lands and livelihoods facing future landscape change. Later in 2023, updated models were integrated with living shoreline suitability maps produced by GIS specialists from Troy University and Louisiana State University (LSU) to identify suitable locations for nature-based restoration overlapping with vulnerable community areas. Simultaneously, the PACIT introduced the Coalition to Restore Coastal Louisiana, a non-profit organization, to Sea Grant as an additional partner for the project, given their expertise with implementing living shorelines in Terrebonne Parish. As Donald Dardar, PACIT Second Chairperson, notes, “Bringing in other partners over the years has helped us with many different projects. They helped us with mapping the area, getting data on our waters, and putting in oyster reefs to protect the land.” Mapping efforts also aligned with Tribal priorities to document ancestral lands as part of the process to gain federal recognition.

While strong foundational relationships are important for sustained co-production, successful partnerships recognize when bringing in new collaborators and ideas can improve a project. Many Tribes face frequent engagement requests from academic, governmental, and nonprofit entities, creating fatigue and skepticism when these requests lack clear benefit to the community (Armitage et al., 2011; Udofia et al., 2017). However, when partnerships have systems to field and vet potential contributors, some interactions lead to meaningful expansion that helps rather than burdens the core relationship. Potential collaborators are often evaluated on their own intentions and expertise, with the PACIT seeking partners whose priorities align with Tribal needs. For example, the Louisiana Universities Marine Consortium (LUMCON) received an award to place additional water level sensors in Louisiana’s bayous to address gaps in its existing sensor network. When a data gap was identified in the Pointe-au-Chien area, the PACIT recognized how local water level information could benefit the Tribe, and Sea Grant helped facilitate the connection with LUMCON to develop a proposal that was ultimately awarded. LUMCON then worked with the PACIT to identify priority sites where the sensors would benefit the Tribe and enhance monitoring efforts for existing and planned restoration and protection projects.

Without strategic evolution, even well-established partnerships can lose momentum between projects, particularly when changing community needs require fresh approaches or expertise. This approach may also smooth over personnel changes when partners need to step back or transition into new roles. Although key partner turnover has been relatively uncommon in the PACIT-Sea Grant partnership, applying an adaptive and responsive framework has helped ensure that strong relationships were maintained rather than weakened when changes did occur. For example, when a personnel transition occurred in the PACIT Resilience Coordinator position, the role was filled by a Tribal staff member who was already familiar with its responsibilities through prior project involvement, enabling continuity of operations throughout the transition. By allowing partnership composition and leadership roles to change alongside shifting priorities and emerging challenges, long-term collaboration can be strengthened.

### Embracing Pluralism

Effective long-term co-production brings knowledge pluralism, a concept that champions the coexistence of seemingly disparate knowledge systems, to bear on the development of research tools and project frameworks (Reid et al., 2021). In the PACIT-Sea Grant collaboration, the development of the Community Resilience Index (CRI)—a tool to assess community preparedness and adaptability to climate and environmental risks—highlighted the importance of embracing pluralism. Sea Grant developed the CRI following the devastating hurricanes in Louisiana, including Katrina in 2005 and Ike in 2008 (Sempier et al., 2010) and it has been implemented successfully in communities and municipalities across the Gulf region since its development. However, the original framework failed to account for how Tribal community resilience is built from cultural practices, unique governance structures, and ongoing environmental stewardship. The CRI inadequately reflected Tribal realities and reinforced the idea that resilience could only be measured through metrics applicable to coastal municipalities with formal government authority.

Acknowledging this limitation, in 2019 the PACIT-Sea Grant partners began co-developing a Tribal Community Resilience Index (TCRI) rooted in pluralism. We conducted workshops with the PACIT and other Indigenous communities in coastal Louisiana to identify gaps and make the index more culturally relevant, such as acknowledging that critical facilities for disaster relief that exist in urban municipalities may not be available in rural Tribal communities. The TCRI acknowledged that resilience in Indigenous contexts is often expressed through kinship networks, cultural continuity, and self-determined land management (Daigle et al., 2025). By allowing multiple ways of knowing to guide the process, the development of the TCRI demonstrated that assessment tools can be both technically rigorous and culturally relevant.

Successful long-term partnerships do not seek to reconcile differences by assimilating one way of knowing into another, which may ignite tensions by assuming one knowledge system is more important or valid than the other. Instead, enduring collaborations embrace pluralism by holding and valuing different perspectives side by side (Bartlett et al., 2012; Reid et al., 2021). As Georgie Ferguson, PACIT Tribal Affairs Liaison, reflects, “I think it’s important that [external partners] witness culture and ways of life that may be different from their own…they should be challenging the ways they’ve always done things…to look at it from others’ perspectives.” By creating space for both Indigenous and academic perspectives to coexist and inform one another, partnerships avoid privileging one knowledge system over another. This approach builds a sense of community and trust, where partners feel heard by seeing their thoughts reflected in the shared work. By keeping both sides invested, pluralism helps to ensure the partnership remains meaningful and beneficial over time.

### Investing Time

Long-term collaboration with Indigenous communities requires more than resources or technical expertise; it requires recognizing time as a core value. The PACIT-Sea Grant partnership embodies this approach through practices that prioritize relationship building and consensus over rigid timelines. For example, our virtual project meetings often begin with relational dialogue, including asking one another about our lives, families, and any ongoing challenges. During Hurricanes like Ida and Francine, we placed more importance on the continued safety and wellbeing of Tribal members than pushing for completion of deliverables. By putting the people first and the project second, we create a sense of community that emphasizes inter-personal connection, building the trust essential for sustained engagement.

Investing time is, therefore, not ancillary to co-production, rather it is a strategy in its own right, ensuring that collaborations remain rooted in relationship rather than driven solely by deliverables. This has shown up in our practice by pursuing planning grants that create more opportunities for us to clarify goals and develop a shared agenda before committing to larger proposals. Project timelines are then built with these long-term goals in mind, such as dedicating time to seek additional funding for follow-up projects. For instance, while developing a living shoreline suitability model for protecting PACIT ancestral spaces, we actively sought funding to take the project through the next phase of site permitting processes. By weaving these larger goals into our timelines, projects can naturally build on one another, sustaining commitment to the partnership that extends well beyond the lifetime of an individual project. We also prioritize careful consideration of all partner viewpoints for meeting when working toward shared objectives. For example, while planning a TEK workshop to support building a living shoreline suitability model, there were differing ideas within the team on how to best capture areas of cultural significance to the PACIT. When it became clear that consensus could not yet be met, additional meetings were planned to allow for continued conversation. These choices reframed time not as a constraint but as an investment in legitimacy and shared ownership of outcomes.

Differences in how time is valued can create tension in academic–Indigenous partnerships if not thoughtfully considered. Despite the recent push towards community engagement, the rewards of academia, such as tenure, grants, and publications, are often tied to fast-paced agendas and rigid deadlines that limit commitment to community work (Nicotera et al., 2011). Indigenous communities, however, may place greater emphasis on building personal relationships that are sustained through long-term engagement and consensus-based decision-making (Adams et al., 2014). We recognize that this mismatch reflects broader academic incentive structures, underscoring the need for institutional change alongside project-level adaptation. At the project level, we have found that academic researchers may narrow this gap by partnering with extension and outreach organizations, like Sea Grant, whose missions are aligned with long-term locally based impact in such areas as community resilience. These organizations often have access to academic researchers while remaining grounded in community concerns, helping to bridge those connections. Additionally, researchers may seek funding from agencies that support community-based resilience efforts. These funders often recognize and value applied projects that create impactful local benefit by prioritizing frequent and long-term engagement. By valuing relational processes and developing shared goals, partners demonstrate respect for Tribal governance structures and cultural practices (Huntington et al., 2011). Investing time becomes not only the medium through which trust is built, but also a tangible demonstration of equity in practice.

### Building Capacity

Our final strategy for sustaining collaborations between academic institutions and Indigenous communities involves moving beyond engagement to invest in building community capacity. In the PACIT-Sea Grant partnership, we have addressed capacity constraints through direct support and appropriate compensation for the PACIT. In 2021, the PACIT created a Resilience Coordinator position partially supported by grant funds including awards received through Sea Grant-PACIT efforts. This person serves as a consistent liaison between the Tribe and external partners, enabling the Tribe to stay informed and engage in research initiatives without relying on overextended volunteer leaders, an all too common reality in many Tribal-academic partnerships. The need for this position grew as the Tribe experienced an influx of potential research partners interested in collaboration after the impact of Hurricane Ida (2021). Once the need was recognized, the position was intentionally included in research proposal budgets. Additionally, small but thoughtful gestures, such as providing meals during meetings, reimbursing travel expenses, and paying guides for site visits, convey respect and support continued participation. During follow-up site visits with volunteer TEK experts to map ancestral areas vulnerable to erosion and sea level rise, lunch was provided to help mitigate financial burdens and acknowledge the value of community members’ time and knowledge. “When they compensate for your time, it makes you feel like what you are doing is important and not taken advantage of” Cherie Matherne, PACIT Resilience Coordinator explains. For communities, like the PACIT, that rely on subsistence livelihoods, even a few hours away from work can have negative economic consequences, making compensation more than symbolic.

While many academic researchers seek to include Indigenous voices, they often underestimate the structural and practical barriers that limit consistent participation. Such barriers include financial constraints (Yua et al., 2022), as many Tribal members contributing to these projects do so on a volunteer basis while juggling responsibilities related to family, subsistence livelihoods, and community leadership. Without realistic strategies for participation, these constraints can also create power-imbalances. Tribal members that are unable to be present may have limited ability to give input, affecting which voices are heard and which projects are prioritized. Addressing these imbalances requires deliberate capacity-building, whether through payment for services (Peterson et al., 2016) or through funding community projects (Satterthwaite et al., 2024). Providing adequate support helps create the conditions under which Indigenous partners can participate meaningfully and incentivizes long-term engagement.

### Bringing the Weave Together

Although we present these strategies independently, their strength lies in how they operate together under real-world pressures. Their interdependence becomes most visible during moments of disruption, when no single approach is sufficient on its own. This convergence was especially evident following Hurricane Ida (2021). As the storm disrupted homes, infrastructure, and livelihoods in Pointe-au-Chien, project timelines were immediately adjusted and extensions negotiated with funders, reflecting flexibility in response to shifting priorities. Rather than pressing forward with deliverables, meetings centered first on community safety, rebuilding needs, and relational continuity, reflecting an intentional investment in time that reinforced trust. The presence of a Resilience Coordinator, supported through prior grant funding, proved critical during this period. This position helped the Tribe to manage incoming research requests, coordinate recovery-related engagement, and maintain continuity working with volunteer leadership, illustrating the importance of capacity-building. In this moment, flexibility, time investment, and capacity-building did not operate independently; together, they sustained the partnership through disruption.

A similar convergence of strategies occurred during the transition from the Community Resilience Index (CRI) to the Tribal Community Resilience Index (TCRI). Recognizing that the original CRI framework inadequately reflected Tribal realities required embracing pluralism and acknowledging institutional blind spots. Multiple workshops were convened to gather input from PACIT and other Indigenous communities, reflecting an investment in time and consensus-based engagement. Compensation for participation and the inclusion of Tribal leadership in decision-making reflected deliberate capacity-building. The resulting tool was not merely adapted but reimagined through evolving partnerships and flexible thinking. This example illustrates how the strategies described above operate collectively to reshape both process and outcome.

The challenges of co-production with local and Indigenous communities have been well documented, from power-imbalances and strained community capacity to constraints related to funding and research timelines (Kolesar et al., 2024; Sokolova et al., 2025; Turnhout et al., 2020). While these challenges remain real, this perspective aims to reframe such instances as opportunities for practitioners to engage more meaningfully. Our experience suggests that these pressures do not necessarily weaken partnerships; rather, they reveal whether collaborative structures are resilient enough to adapt. By weaving flexibility, pluralism, time investment, evolving partnerships, and capacity-building together, the PACIT–Sea Grant collaboration has been able to translate moments of strain into opportunities for institutional learning and strengthened relationship continuity. This has led to local infrastructure improvements such as an improved bulkhead and dock area that demonstrate tangible benefits for the community (Fig. 1). In addition, outcomes such as the TCRI, guidance documents, and peer-reviewed publications are not endpoints, but evolving tools embedded in a longer relational process. These products have been applied through workshops, infrastructure planning, and follow-on projects, and continue to shape future resilience efforts within the PACIT and beyond. In this way, co-produced outputs function as living components of an enduring partnership, demonstrating how sustained collaboration can support both community resilience and institutional transformation.

## Conclusion

Authentic collaboration with Indigenous communities requires sustained commitment that often extends beyond individual projects and grants. The challenges that are often encountered in these partnerships, such as differing worldviews, capacity constraints, and shifting priorities, are opportunities to strengthen relationships through deliberate practice.

Throughout more than a decade of collaboration between PACIT and Sea Grant, five strategies have been essential for longevity: Maintaining Flexibility, Evolving Partnerships, Embracing Pluralism, Investing Time, and Building Capacity. While this case study demonstrates successful long-term collaboration, the strategies we identify emerged from our coastal Louisiana context with specific institutional and community features. How well our approach transfers to other research settings will depend on local contexts, resources, and priorities. Despite potential differences in setting, the importance and success of these strategies have been documented across many independent research projects (Hill et al., 2020; Reid et al., 2021; Yua et al., 2022), and we conclude that when woven together, they can also support sustained academic-Indigenous partnerships.

This perspective suggests that successful co-production depends less on the ideal starting conditions and more on sustained commitment to equitable partnerships that can withstand both external pressures and internal complexities. By analyzing this enduring PACIT-Sea Grant collaboration, we can better understand how partnerships in co-production evolve and provide guidance on how they can be maintained and strengthened over time.
